# Expedited research and approval of medicines for drug-resistant bacterial infections in children

**DOI:** 10.2471/BLT.25.295493

**Published:** 2026-05-27

**Authors:** Phoebe CM Williams, Gary J Noel, Sumati Nambiar, Seamus O’Brien, David L Paterson, Julia A Bielicki, Michael Sharland, John S Bradley

**Affiliations:** a Sydney Institute for Infectious Diseases, University of Sydney, Sydney, Australia.; b Great Falls, Virginia, United States of America (USA).; c Child Health Innovation and Leadership Department, Johnson and Johnson, Raritan, USA.; d Global Antibiotic Research and Development Partnership, Geneva, Switzerland.; e University of Queensland Centre for Clinical Research, Royal Brisbane and Women's Hospital, Herston, Australia.; f Centre for Neonatal and Paediatric Infection, City St George's University of London, London, England.; g University of California San Diego, School of Medicine, Department of Paediatrics, Division of Infectious Diseases, 3020 Children’s Way, MC 5041, San Diego, California, CA 92123, USA.

## Abstract

Multidrug-resistant pathogens cause severe infections with substantial morbidity and mortality in neonates, infants and children, as they do in adults. However, the approval of therapies for multidrug-resistant infections in paediatric patients often lags years behind approval for adults, which creates an untenable situation for people who care for affected children. Many issues contribute to the current unacceptably long period between adult and paediatric new drug approval, including the requirement for sequential age group enrolment in clinical trials, the types of regulatory studies required and a lack of alignment between regulatory authorities. To streamline paediatric drug development, more efficient approaches to investigational and approval processes that meet acceptable safety and efficacy metrics are required. For products that address an urgent unmet need in children, approval should be based primarily on matching drug exposure to levels in adults and on extrapolating safety and efficacy data from adults, where appropriate, rather than on requiring clinical trials in all paediatric age groups for each approved indication in adults. In addition, real-world and post-approval data could be used to support early, limited drug approval for paediatric patients. However, there are economic challenges in developing anti-infective drugs for paediatric populations and increased funding is needed to ensure that neonates, infants and children globally have timely access to safe, effective and life-saving therapies.

## Résumé

Les agents pathogènes multirésistants provoquent des infections graves associées à une morbidité et à une mortalité élevées chez les nouveau-nés, les nourrissons et les enfants, tout comme chez les adultes. Cependant, l’autorisation de traitements contre les infections multirésistantes chez les patients pédiatriques accuse souvent un retard de plusieurs années par rapport à celle accordée aux médicaments destinés aux adultes, ce qui crée une situation intenable pour les personnes qui ont à charge les enfants touchés. De nombreux facteurs contribuent au délai actuellement inacceptable entre l’autorisation d’un nouveau médicament chez l’adulte et chez l’enfant, notamment l’obligation de recruter les groupes d’âge de manière séquentielle dans les essais cliniques, les types d’études réglementaires requises et un manque de coordination entre les autorités réglementaires. Afin de rationaliser le développement des médicaments pédiatriques, il est nécessaire de mettre en place des approches plus efficaces pour les processus d’investigation et d’autorisation afin que ceux-ci répondent à des critères acceptables en matière de sécurité et d’efficacité. Pour les produits destinés à répondre à un besoin urgent non satisfait chez l’enfant, l’autorisation devrait reposer principalement sur la mise en correspondance de l’exposition au médicament et des niveaux observés chez l’adulte ainsi que sur l’extrapolation des données de sécurité et d’efficacité issues des études chez l’adulte, le cas échéant, plutôt que sur l’exigence d’essais cliniques dans tous les groupes d’âge pédiatriques pour chaque indication approuvée chez l’adulte. En outre, des données issues de la pratique clinique et post-autorisation pourraient servir à étayer une autorisation précoce et limitée de ces médicaments pour les patients pédiatriques. Toutefois, la mise au point de médicaments anti-infectieux destinés aux populations pédiatriques pose des défis économiques. Dès lors, un financement accru est nécessaire pour garantir que les nouveau-nés, les nourrissons et les enfants du monde entier aient accès en temps opportun à des traitements sûrs, efficaces et vitaux.

## Resumen

Los patógenos multirresistentes causan infecciones graves con una morbilidad y mortalidad considerables en neonatos, lactantes y niños, al igual que en las personas adultas. Sin embargo, la aprobación de tratamientos para infecciones multirresistentes en pacientes pediátricos suele producirse años después de su aprobación para adultos, lo que genera una situación insostenible para quienes atienden a los niños afectados. Numerosos factores contribuyen al periodo, actualmente inaceptablemente largo, que transcurre entre la aprobación de nuevos medicamentos para adultos y para pacientes pediátricos, entre ellos la exigencia de una inclusión secuencial por grupos de edad en los ensayos clínicos, los tipos de estudios regulatorios requeridos y la falta de armonización entre las autoridades reguladoras. Para agilizar el desarrollo de medicamentos pediátricos, se requieren enfoques más eficientes para los procesos de investigación y aprobación que cumplan criterios aceptables de seguridad y eficacia. En el caso de productos destinados a cubrir una necesidad urgente no satisfecha en la población infantil, la aprobación debería basarse principalmente en alcanzar niveles de exposición al medicamento comparables a los observados en adultos y en extrapolar, cuando proceda, los datos de seguridad y eficacia obtenidos en adultos, en lugar de exigir ensayos clínicos en todos los grupos de edad pediátrica para cada indicación aprobada en adultos. Además, los datos procedentes de la práctica clínica real y los obtenidos tras la aprobación podrían utilizarse para respaldar una aprobación temprana y limitada de medicamentos para pacientes pediátricos. No obstante, existen desafíos económicos para el desarrollo de medicamentos antiinfecciosos destinados a la población pediátrica, y se necesita una mayor financiación para asegurar que los neonatos, lactantes y niños de todo el mundo tengan acceso oportuno a tratamientos seguros, eficaces y que salvan vidas.

## ملخص

إن مسببات الأمراض المقاومة للعقاقير المتعددة تؤدي إلى حالات عدوى خطيرة ذات معدلات عالية من الإصابة بالأمراض والوفيات لدى حديثي الولادة، والرضع، والأطفال، كما هو الحال لدى البالغين. ومع ذلك، فإن الموافقة على علاجات العدوى المقاومة للعقاقير المتعددة لدى الأطفال، غالبًا ما تتأخر لسنوات عن الموافقة عليها لدى البالغين، مما يخلق وضعًا صعبًا للغاية بالنسبة للأشخاص الذين يرعون الأطفال المصابين. تسهم العديد من العوامل في طول الفترة الحالية غير المقبولة بين الموافقة على الأدوية الجديدة للبالغين والأطفال، بما في ذلك اشتراط التسجيل المتسلسل للفئات العمرية في التجارب الإكلينيكية، وأنواع الدراسات التنظيمية المطلوبة، وعدم التنسيق بين الهيئات التنظيمية. وحتى تتسم عملية تطوير الأدوية للأطفال بالسلاسة، يجب اتباع أساليب أكثر فعالية في عمليات الاستقصاء والموافقة، بما يضمن الوفاء بمعايير السلامة والفعالية المقبولة. بالنسبة للمنتجات التي تلبي حاجة ملحة غير مستوفاة لدى الأطفال، يجب أن تستند الموافقة في المقام الأول إلى مطابقة مستويات التعرض للعقار مع مستويات البالغين، واستقراء بيانات السلامة والفعالية من البالغين، عند الحاجة، بدلاً من اشتراط إجراء تجارب إكلينيكية على جميع الفئات العمرية للأطفال لكل استخدام مُعتمد لدى البالغين. إضافةً إلى ذلك، يمكن استخدام بيانات الواقع العملي وبيانات ما بعد الموافقة لدعم الموافقة المبكرة والمحدودة للأدوية لمرضى الأطفال. مع ذلك، توجد تحديات اقتصادية في تطوير العقاقير المضادة للعدوى للسكان من الأطفال، وهناك حاجة إلى زيادة التمويل لضمان حصول حديثي الولادة، والرضع، والأطفال في جميع أنحاء العالم على علاجات آمنة وفعالة ومنقذة للحياة في الوقت المناسب.

## 摘要

多重耐药病原体不仅会在新生儿、婴儿和儿童中，也会在成人中导致严重感染，且致病率和致死率都相当高。然而，针对儿科患者的抗多重耐药菌感染疗法的审批往往比成人疗法滞后多年，从而让需要照护此类患儿的人员陷入难以维持的局面。导致当前成人和儿科用新药审批时间之间存在无法接受的差距的原因有很多，包括临床试验需按年龄组依次入组、监管机构对研究类型有特定要求以及监管当局之间缺乏协调统一。如要简化儿科药物研发流程，需要采取更有效的方法来优化试验和审批流程，并达到可接受的安全和疗效指标。审批可解决儿童紧急未满足需求的产品时，若适当，应主要基于药物暴露水平是否与成人相匹配，并根据成人数据外推安全性和疗效，而非要求逐一针对已获批的成人适应症，在各年龄组儿科患者之间开展临床试验。此外，真实世界数据和审批后数据可用于支持提早、有限制地审批儿科患者用药。但是，为儿科患者群体研发抗感染药物还会面临多重经济挑战，需加大资金投入以确保全球新生儿、婴儿和儿童能够及时接受可挽救其生命的安全有效疗法。

## Резюме

Патогены, обладающие множественной лекарственной резистентностью, вызывают тяжелые инфекции со значительными показателями заболеваемости и смертности среди новорожденных, младенцев и детей старшего возраста, в равной степени как и среди взрослых. Однако одобрение схем лечения инфекций со множественной лекарственной резистентностью для пациентов детского возраста отстает на годы от решения этого же вопроса для взрослых, что создает неприемлемую ситуацию для лиц, осуществляющих уход за больными детьми. Причин такого недопустимо длительного периода между одобрением новых препаратов для применения у взрослых и детей множество, в том числе это требование привлечения последовательных возрастных групп к участию в клинических исследованиях, требуемые типы регуляторных исследований, а также недостаточная согласованность работы регулирующих органов. Чтобы упростить разработку лекарств для детей, необходимы более эффективные подходы к процессам исследований и одобрения, которые отвечают приемлемым показателям безопасности и эффективности. Для продукции, отвечающей острой неудовлетворенной потребности в медицинской помощи детям, процедура одобрения должна опираться главным образом на сопоставление воздействия препарата с уровнями, достигаемыми у взрослых пациентов, и на экстраполяцию данных по безопасности и эффективности, собранных для взрослых, когда это уместно, а не на требование обязательного проведения клинических испытаний во всех возрастных группах пациентов-детей для каждого показания к применению, одобренного для взрослых. Кроме того, данные реальной клинической практики и пострегистрационного применения можно использовать для обоснования раннего ограниченного одобрения тех или иных препаратов в случае применения у пациентов детского возраста. Однако разработка противоинфекционных препаратов для педиатрических популяций сталкивается с проблемами экономического характера, и необходимо увеличение финансирования для того, чтобы новорожденные, младенцы и дети старшего возраста во всем мире гарантированно имели своевременный доступ к безопасному и эффективному лечению, спасающему жизни.

## Introduction

Globally, there is an urgent need for effective antimicrobial therapy against multidrug-resistant bacteria for all paediatric age groups, especially neonates who experience the highest morbidity and mortality.^1^^,^^2^ Pathogens causing substantial morbidity and mortality in neonates, infants and children include both Gram-positive bacteria, such as methicillin-resistant *Staphylococcus aureus* and vancomycin-resistant *Enterococcus*; and Gram-negative bacteria, such as *Pseudomonas* spp., *Klebsiella* spp., *Escherichia coli*, *Acinetobacter* spp., *Burkholderia* spp. and *Stenotrophomonas* spp.^3^

Although the need for novel therapies to address the burden of antimicrobial resistance in children has been recognized as a priority, no accepted expedited development pathway exists. Therefore, researchers, sponsors (i.e. usually pharmaceutical companies who discover and investigate new therapies for clinical use), regulators and families involved in drug development should prioritize the unmet needs of neonates, infants and children and study new antimicrobial drugs in a transparent, efficient and collaborative manner.

The urgency of accelerating paediatric antibacterial drug development has been reflected in recent initiatives by the World Health Organization (WHO), including the paediatric drug optimization process;^4^ updated target product profiles for guiding the development of new antibacterial agents, particularly for children;^5^ and updated global research priorities for paediatric clinical trials.^6^ These initiatives emphasize regulatory harmonization requirements, prioritization of key antibacterials for children and globally coordinated development pathways. This manuscript builds on these WHO-led programmes by outlining the practical challenges faced in expediting the provision of novel antibacterial agents for neonates and children and by proposing strategies for meeting these challenges.

## Delays in access

Currently, once a new therapy has been approved for adults, it may be several years before approval is granted for children.^1^ Moreover, there are often additional delays for neonates and young infants, who are usually studied after older children. Despite efforts by regulatory authorities and WHO to promote the development of paediatric antibacterials, between 2000 and 2020 it took up to 7.5 years for a new parenteral antibacterial drug approved for use in adults to be approved for children by the United States Food and Drug Administration; furthermore, during those two decades, this delay did not get shorter.^7^^,^^8^ Delays in completing the trials required by regulators arise from difficulty of enrolling children and the lack of experienced, efficient, paediatric clinical trial networks. Further, the regulatory safety and efficacy requirements imposed on relatively safe classes of antibacterials seem unnecessary for all indications in all paediatric age groups, particularly when substantial safety and efficacy data are available for adults or older paediatric age groups.^9^

Clinicians facing the difficult decision of whether to prescribe new antibacterials that have been approved for multidrug-resistant infections only in adults are also confronted by a lack of access to safety and efficacy data from paediatric drug trials. Often these data are not shared publicly after trial completion, or are not reported in the peer-reviewed literature until well after the agents have been approved for adults.^10^ However, making these data available as soon as possible for investigators and regulators can be challenging for pharmaceutical sponsors.

Traditionally, the evaluation of new antimicrobial drugs for clinical use has been the responsibility of sponsors who are required by regulators in the European Union and United States of America to assess their product in all paediatric age groups, unless specific waiver criteria are met. Paediatric trials are more costly than adult trials and are often financially borne by antibacterial development bodies such as pharmaceutical companies, governments, professional societies and foundations, often in public–private partnerships. Pharmaceutical companies, which have historically played a large role in drug development, are unlikely to recover these costs by selling drugs that are used to treat only rare multidrug-resistant infections.^11^ Moreover, the sites conducting paediatric drug trials in cooperation with sponsors often do so at a financial loss over the duration of the study and rely on the, often voluntary, efforts of paediatricians, nurses and other care-providers to meticulously collect and report the trial data required by regulators.

## Global inequalities

Access delays of new drugs are particularly concerning for low- and middle-income countries, where the need is the greatest, especially for neonates and young infants. Conducting clinical trials in these resource-constrained settings is particularly challenging given the high clinical workload, the limited research experience in some instances, and the complex ethical considerations when involving children in trials, particularly where available therapies are inadequate.^12^^–^^15^

## Strategies for expediting development

Stakeholders may accept greater risks during investigations into new therapies if the targeted infection is severe and the consequences of inadequate treatment are unacceptable. Paediatric drug development could adopt a more efficient and tailored approach if nonclinical and adult safety data and information on the safety of similar products in children are considered.^7^ Then, when the benefit–risk balance is acceptable and the need is urgent, regulatory authorities would be more likely to accept a streamlined development programme for expedited approval. The following sections outline key opportunities for improving the efficiency of paediatric antibacterial drug development, from overarching principles to practical implementation strategies (Box 1, Table 1).

Box 1 **Strategies for expediting paediatric antibacterial drug development**

***Routinely include adolescents in clinical trials***

When sufficient safety data for adults are available to assess the risks and benefits of a treatment, adolescents should be enrolled in adult phase-2 or −3 trials or in a parallel cohort.

***Extrapolate safety data from adult studies***

Extrapolating safety data from adults to children can help reduce the trial size required for certain products or age groups, particularly when the drug being studied is a member of a known and well-studied antibiotic class (e.g. β-lactam–β-lactamase inhibitor combinations).

***Streamline pharmacokinetic data collection***

Obtain pharmacokinetic data from studies in which investigational products are given in addition to standard-of-care treatment, for any indication.

***Use noncomparative trials***

Consider using single-arm, open-label studies in children for licensing and approval purposes rather than large, standard studies with comparator treatments, for which recruitment is difficult.

***Strengthen global paediatric trial networks***

Fund public–private partnerships that support global, paediatric, clinical trial networks investigating novel antibacterial drugs in neonates and children.

**Table 1:** Strategies and actions for expediting the development of new agents to treat multidrug-resistant infections in children and neonates

Strategy	Action	Advantages	Challenges and risks	Priority for implementation
Global regulatory alignment and harmonization	Align requirements across regulatory authorities and policy-making bodies	(i) Reduces the risk of duplication and unnecessary additional trials; (ii) streamlines drug development; and (iii) improves global access to new drugs	Requires coordination across jurisdictions that may have different regulatory priorities	High
Expanded paediatric clinical trial networks	Develop sustainable, well-funded, international networks to support efficient trial implementation in both resource-rich and resource-constrained health-care settings	(i) Makes trial recruitment easier; (ii) builds capacity for conducting trials; (iii) enhances data quality; and (iv) ensures clinical findings are generalizable across all paediatric populations	Requires substantial, sustainable funding and coordination to ensure gains in capacity-building are maintained	High
Noncomparative trial designs	Conduct single-arm, open-label pharmacokinetic and safety studies rather than small, underpowered comparative trials	(i) Increases trial feasibility; (ii) avoids inefficient trials; and (iii) reduces delays in implementation	Comparative safety data are limited	High
Extrapolation of efficacy data from adults	Use adult efficacy data to expedite paediatric drug approval	(i) Avoids unnecessary efficacy trials; (ii) widely accepted by regulators; and (iii) efficient	May not be appropriate for all infections, particularly where the disease pathophysiology differs between adults, children and neonates	High
Extrapolation of safety data from adults	Use existing adult and paediatric safety data, especially for known drug classes (e.g. β-lactam–β-lactamase inhibitor combinations)	(i) Reduces the need for large paediatric trials; and (ii) enables earlier approval	Close postmarketing monitoring is required to detect unforeseen toxicity risks, particularly for neonates	High
Simultaneous pharmacokinetic investigations across paediatric age groups	Conduct pharmacokinetic studies in multiple paediatric age groups concurrently (e.g. using weight-banded dosing) rather than sequentially	(i) Avoids delays inherent in age de-escalation; and (ii) accelerates neonatal access to drugs	(i) Requires careful safety oversight; and (ii) may not be appropriate for novel drug classes	High
Inclusion of adolescents in adult trials	Enrol adolescents in phase-3 adult trials, when appropriate, to enable earlier drug labelling for children	(i) Enables near-simultaneous approval for adolescents and adults; and (ii) reduces trial duplication and the need for slow age de-escalation trials	Requires the alignment of regulatory and ethical frameworks, as well as the willingness of site investigators to enrol younger cohorts	High
Expedited conditional approval pathways	Allow rapid drug approval for conditions with a high unmet need for novel antimicrobials and support approval through postmarketing studies	Enables timely access to life-saving therapies	(i) There may be a higher level of uncertainty about safety and efficacy at approval; and (ii) strong post-licensing, pharmacovigilance, surveillance systems must be in place	High
Strengthened data systems and standardization	Improve data collection tools, surveillance systems and the standardization of definitions of the populations studied, study inclusion and exclusion criteria, clinical and laboratory assessments, pharmacometrics for drug exposure, adverse events, and short- and long-term treatment outcomes	(i) Improves data quality; (ii) facilitates both trial implementation and postsurveillance real-world data collection; and (iii) enables pooled analyses	Requires substantial infrastructure investment and coordination	Moderate
Standardized contracts and protocols	Use harmonized contracts, trial protocols and operating procedures across trial sites and regions	(i) Reduces delays in site activation and study commencement; and (ii) improves transparency and efficiency	May be difficult to implement across diverse legal and institutional systems	Moderate
Real-world data use	Use observational data, including data attained from off-label prescribing, to complement clinical trial data on safety and effectiveness	(i) Reflects real-world drug use; and (ii) increases pre- and post-approval evidence generation	(i) Information on off-label use may not always be published; (ii) data available for novel agents may be limited; and (iii) prescribing bias and confounding is possible in certain patient populations	Moderate

### Trials for expedited approval

The prime purpose of paediatric studies of new drugs is usually to address pharmacokinetics and safety. For pharmacokinetics assessments, a study design whereby a single dose or multiple doses of the investigational product are given in addition to standard-of-care therapy for any indication could be considered adequate. This approach will expedite development by not limiting pharmacokinetic assessments in children to approved adult indications, which is particularly important given the existing global variation in approval criteria for adults, such as the body site-specific indications used by the United States Food and Drug Administration and the pathogen-based indications used by the European Medicines Agency.^16^^,^^17^ Safety assessments, in contrast, may be confounded by concurrent standard-of-care antibacterial therapy. Potential drug–drug interactions must be evaluated, including by in vitro assessment, where relevant. 

Comparative trials are often inefficient, unlikely to generate robust safety data in small paediatric samples and may delay access. In some cases, single-arm, open-label, noncomparative pharmacokinetic and safety studies should be considered as the basis for approval, as is the norm for other paediatric infectious disease trials, such as for tuberculosis and human immunodeficiency virus (HIV) infection.^4^ Although the inclusion of a comparator arm has advantages, obtaining robust comparative safety data is difficult when only a small number of children are enrolled. The utility, interpretability and cost–effectiveness of larger, more complicated, comparative studies that assess both efficacy and safety is questionable, particularly where recruitment is difficult (e.g. for multidrug-resistant infections) and the primary goal is to describe pharmacokinetics and safety. In these circumstances, greater use should be made of the safety data for children available from previous trials on comparator antibacterial agents and from the published literature rather than requiring comparative trials for all products.^18^

### Simultaneous investigation

Accurate pharmacokinetics data and an understanding of drug exposure are essential before conducting studies of clinical efficacy, but the traditional approach of completing pharmacokinetic studies sequentially in decreasing paediatric age cohorts delays the access to novel antimicrobials.^18^ This lengthy process may not be needed, especially for classes of antimicrobial drug that have predictable pharmacokinetics and a well-established safety profile. Where appropriate, simultaneous pharmacokinetics investigations could be conducted across multiple paediatric age groups using weight-banded dosing, which is consistent with the WHO recommendations and practices applied in other therapeutic areas, such as the treatment of HIV infection.^4^ However, although some drugs can be investigated simultaneously in all paediatric age groups, including neonates, for others, particularly new classes of drug, data from older children should be reviewed before investigations are conducted in more vulnerable and fragile paediatric age groups.^19^

### Extrapolating safety data

A key principle underpinning more efficient paediatric drug development is the appropriate extrapolation of safety data from adults to children. Extrapolation can help optimize the trial size required for certain products or age groups, particularly when the drug under investigation is a member of a known and well-studied class of antibacterials, such as β-lactam–β-lactamase inhibitor combinations.^18^ Small clinical trials involving four to six subjects per age group could be used to supplement the extrapolation of efficacy data from adults to children, thereby helping achieve approval for children and neonates. There may be some concerns about using this approach for new classes of antimicrobial with less information available for either adults or children or with known toxicities in children (e.g. tetracyclines and fluoroquinolones).^20^ However, risks could be mitigated and safety in children could be established through post-approval studies or pharmacovigilance.^19^^,^^21^

Extrapolating safety data from adults should consider any off-target effects of concern and the potential for age-specific or exposure-specific toxicities. For some drugs, the risks may be greater in neonates and young infants than in older children or adults; for example, central nervous system exposure may be increased by the immaturity of the blood–brain barrier and the immature neonatal brain may be more susceptible to toxicity.^17^ In these circumstances, additional data should be collected. However, European Medicines Agency guidelines acknowledge that safety profiles in adults and children are expected to be similar for similar systemic exposures, which supports the extrapolation of safety data from adults to children.^17^ In addition, the International Council for Harmonisation’s 2024 E11A paediatric extrapolation supports a structured and continuum-based approach to the extrapolation of safety data.^22^ This guidance also recommends using modelling and targeted data collection to help address uncertainty and devise more efficient paediatric trial designs.

### Extrapolating efficacy data

Paediatric drug development could also be streamlined by extrapolating efficacy data from adults to children for matched drug exposure. Modelling or simulating the relationships between drug exposure and clinical or microbiological outcomes could reduce the number of children that need to be enrolled in trials to validate exposure levels, thereby expediting the approval of new drugs. Nevertheless, paediatric antibacterial trials should be designed primarily to assess pharmacokinetics and safety, rather than efficacy. For most infectious diseases, adult exposure data, along with associated microbiological and clinical efficacy data, can be used to support paediatric drug approval. However, pathogen characteristics, their pathophysiology and the clinical presentation must be considered to address important differences between adults and children or between paediatric cohorts.^16^^,^^17^ For example, bacterial sepsis is specific risk for neonates and young infants because of differences in clinical and microbiological responses to particular drug exposure levels, thereby making it difficult to reliably extrapolate data from adults or older children.^23^

### Adolescents in adult trials

When adequate data from early phase studies in adults are available to assess the risks and benefits of a new drug, efforts should be made to enrol adolescents in adult phase-3 trials or in parallel cohorts. This approach is consistent with regulatory guidance, has been successfully implemented in other therapeutic areas and can lead to the simultaneous approval and labelling of a new drug for use in both adults and children as young as 12 years of age.^16^^,^^17^ Moreover, the early availability of data on adolescents can enable studies in younger children to start sooner.

### Neonates

Neonates, including premature neonates, and infants younger than 3 months require additional considerations due to their particular physiology and to the high unmet clinical need for novel antimicrobials in this age group. The current substantial delay in addressing this population’s needs is unacceptable and results in a high number of unnecessary deaths.^1^ During the first weeks of life, the pharmacokinetics and safety of antimicrobial drugs can be extensively affected by rapidly evolving differences in the distribution of drugs between tissues and by the maturational development of renal and hepatic clearance.^24^

In neonates, assessment of toxicity is challenging because physical examination is limited and blood sampling difficult. In addition, the long-term impact of adverse events following drug exposure, which may not be apparent at the time of administration, may be difficult to determine. Further, the immaturity of the immune system during the first few months of life can also affect the assessment of efficacy because clinical outcomes depend on both antimicrobial drug activity and the host immune response.^23^ Finally, effectiveness may be influenced by obstacles to drug administration, including the limited availability of age-appropriate formulations and variations in drug delivery, particularly for critically ill neonates.

Consequently, pharmacokinetics, safety, formulation, dosing and efficacy of drugs must be addressed across a wide range of gestational and chronological ages. The feasibility of different administration methods must also be considered to ensure optimal uptake and real-world effectiveness. For drug classes for which substantial safety and efficacy data are available for older children and adults, such as β-lactam–β-lactamase inhibitor combinations, approval could be based on data from fewer neonates. However, this approach might not be appropriate for new molecular entities.

### Expedited approval pathways

Drug development programmes for specific paediatric groups could be streamlined, similar to the limited approvals granted for adults, provided safety and efficacy limitations in children is clearly understood.^18^ Additional evidence could be systematically collected from publications, postmarketing reports and databases, with post-approval safety reporting helping to address the initial data gaps. Further, modelling and simulation, extrapolation and the use of real-world data are critical for complementing data from clinical trials on the benefit–risk balance of a product. Although real-world data have limitations, they provide useful information on safety, rare adverse events, real-world effectiveness and dosing patterns in clinical practice. As novel agents may be used infrequently in early post-approval settings, real-world data have to be interpreted with caution and their ability to generate robust or generalizable evidence may be limited.^20^ Furthermore, the risks and benefits of expedited approval should be carefully considered for high-risk populations, such as neonates. Targeted safety evaluations and robust postmarketing surveillance are necessary to ensure that access to life-saving therapy is balanced by careful and ongoing risk assessment.

### Off-label antimicrobial use

The off-label use of new antimicrobial drugs in the period between adult and paediatric approval can provide an important source of real-world evidence that complements structured data collection through clinical trials and postmarketing surveillance. Clinical information about dosing and the risks and benefits of off-label use is critical before paediatric approval, particularly when children cannot be enrolled in a clinical trial for these agents and no other treatments exist. 

### Regulatory alignment

Regulatory requirements for paediatric drug development should be harmonized between the European Medicines Agency, the United States Food and Drug Administration and other regulatory authorities to streamline global drug development.^18^ Aligning paediatric investigational protocols would also facilitate timely planning and execution. Ideally, high-quality, paediatric data generated in one region should support regulatory approval in other regions. Existing pathways for regulatory national cooperation should be expanded, especially where there is an urgent need.^17^

The development of international, standardized, paediatric drug investigational protocols for commonly studied indications (e.g. skin infections, urinary tract infections and pneumonia), as often used in adult drug development, could help streamline regulatory processes in individual countries and make the approval and conduct of paediatric clinical trials more efficient. In addition, standard protocols would facilitate pooled data analysis, thereby increasing understanding of the safety, efficacy and pharmacokinetics of drugs across all paediatric age groups. As countries beyond the European Union or the United States may submit paediatric approvals early, regulatory alignment before paediatric trials is critical to ensure the studies meet the regulators’ requirements.^19^

### Early submission of trial data

Given that the approval and labelling of some novel antimicrobials (e.g. bedaquiline and HIV drugs) is carried out in a sequential, age-stratified manner, it is important that the data from older age cohorts of children used to support approval are submitted as soon as possible, because early submission can be very effective for ensuring drug labelling is updated for older children while clinical trials in younger age groups, including neonates, are still ongoing.^6^^,^^9^ Although multiple submissions of data may be burdensome for sponsors and regulatory agencies, approval and labelling updates can be timelier and, in any case, multiple submissions are needed to ensure the appropriate use of some new antibacterials.^18^ Moreover, regulators considered this an acceptable approach.

### Expanding clinical trial networks

In the United States, there are no successful, free-standing, paediatric trial networks for investigating drugs for regulatory approval. Setting up paediatric investigation sites is costly and time-intensive, often repeated for each new drug developed. For example, some phase-2 and -3 studies of new paediatric antimicrobial drugs required up to 50 clinical trial sites worldwide, and it takes years to identify, contract and deliver training at the sites. For most antimicrobial drug trials, the average number of subjects enrolled at each site is small. In our experience with facilitating studies involving children, two or fewer children may be enrolled at each site and as many as half the sites involved may not enrol a single patient.^25^ Consequently, trial costs per evaluable child can be substantially greater than the cost per adult enrolled in comparable trials, which reflects the complexity of, and resources required by, paediatric studies. Rather than being a profitable investment, these trials are usually a financial liability that the sponsor must accept to comply with the drug development programme required.

In contrast, there are paediatric clinical trial networks outside the United States, such as the African–European SNIP-AFRICA collaboration;^26^ the Paediatric European Network for the Treatment of AIDS and Infectious Diseases;^27^ and initiatives by the Global Antibiotic Research and Development Partnership.^28^ However, attempts to create sustainable paediatric trial site networks for antibacterial drug development have been hampered by limited support for full-time academics and health workers to be involved, and the high cost and resources required to recruit and sustain the personnel needed. Although funding mechanisms for clinical trial networks exist globally, securing reliable, sustainable funding (e.g. from sponsors, governments or nongovernmental organizations) remains a major barrier.

Public–private collaborations can play a role in establishing sustainable global paediatric trial networks, but adequate funding for capacity-building and the establishment of networks is essential, particularly where unmet needs for novel antimicrobials are greatest. In addition, the efficient implementation of clinical trials and the generation of reliable data depend on strengthening data surveillance by providing adequate funding and infrastructure and on improving the standardization of key variables, such as definitions of the populations studied, inclusion and exclusion criteria, pharmacometrics for drug exposure, clinical and laboratory assessments, adverse events, and short- and long-term treatment outcomes.

### Trial site contracting and funding

The clinical trial contracting process is often complex and time-consuming and varies greatly across sites and regions, thereby contributing to delays in activating sites and initiating trials. In many resource-constrained health-care settings, the limited availability of dedicated research staff to assist in executing contracts can delay the implementation of trials where they are most needed. Contracts are important because they specify the obligations of sponsors, investigators and sites and define publication and intellectual property rights. However, their implementation could be simplified by the creation of a standardized contract to be shared with all research sites, which would increase transparency and promote dialogue between sites to ensure a successful, collaborative relationship. Standardization could also expedite the research process and the publication of results. Information on budgeting should be transparent, standardized and shared between sites and sponsors or clinical research organizations to ensure that trials are conducted appropriately and no site makes a profit or loss.

### Engaging community stakeholders

In addition to sponsors and regulators, community stakeholders, such as trial investigators, health workers and parents, should be fully engaged in the approval and labelling of novel antibacterials to treat drug-resistant infections. Early engagement of stakeholders, starting at protocol design, will help ensure understanding of the trial, smooth enrolment and successful completion. Early engagement has been shown to make recruitment easier, to ensure that study procedures and the overall conduct of the trial are acceptable in a paediatric setting, and clarifies the risks and benefits associated with a child’s participation.^29^^,^^30^

## Conclusion

All stakeholders involved in promoting the health of neonates, infants and children need to collaborate to address the challenges of paediatric drug development. The study populations required for paediatric drug trials could be reduced by ensuring pharmacokinetic and safety investigations of new antibacterial drugs are performed efficiently, and by designing clinical trials to take advantage of extrapolated efficacy data from adult trials, where appropriate. In addition, real-world and post-approval data could be used to support early, limited drug approval to enable prompt paediatric access to critically needed antibacterials active against multidrug-resistant pathogens. Nevertheless, we acknowledge that there are economic challenges in developing anti-infective drugs for all paediatric populations. Increased funding is needed from global governments, industry and philanthropic sources, along with statutory financial incentives for novel paediatric antibacterials, to ensure that neonates, infants and children in both well-resourced and resource-constrained health-care settings have timely access to safe, effective and often life-saving therapies.
